# Evaluation of DNA Vaccine Candidates against Foot-and-Mouth Disease Virus in Cattle

**DOI:** 10.3390/vaccines11020386

**Published:** 2023-02-07

**Authors:** Michael Puckette, Benjamin A. Clark, José Barrera, John G. Neilan, Max V. Rasmussen

**Affiliations:** 1 U.S. Department of Homeland Security Science and Technology Directorate, Plum Island Animal Disease Center, P.O. Box 848, Greenport, NY 11944, USA; 2 Oak Ridge Institute for Science and Education, Plum Island Animal Disease Center Research Participation Program, P.O. Box 848, Greenport, NY 11944, USA; 3 Leidos, Plum Island Animal Disease Center, P.O. Box 848, Greenport, NY 11944, USA

**Keywords:** minicircle, foot-and-mouth disease virus, plasmid, DNA, vaccine, cattle, in vivo

## Abstract

We evaluated four DNA vaccine candidates for their ability to produce virus-like particles (VLPs) and elicit a protective immune response against Foot-and-mouth disease virus (FMDV) in cattle. Two traditional DNA plasmids and two DNA minicircle constructs were evaluated. Both the pTarget O1P1-3C plasmid and O1P1-3C minicircle encoded a wild-type FMDV 3C protease to process the P1-2A polypeptide, whereas the O1P1-HIV-3C^T^ minicircle used an HIV-1 ribosomal frameshift to down-regulate expression of a mutant 3C protease. A modified pTarget plasmid with a reduced backbone size, mpTarget O1P1-3C^LT^, used a 3C protease containing two mutations reported to enhance expression. All constructs produced mature FMDV P1 cleavage products in transfected cells, as seen by western blot analysis. Three constructs, O1P1-3C minicircles, pTarget O1P1-3C, and mpTarget O1P1-3C^LT^ plasmids, produced intracellular VLP crystalline arrays detected by electron microscopy. Despite VLP formation in vitro, none of the DNA vaccine candidates elicited protection from clinical disease when administered independently. Administration of pTarget O1P1-3C plasmid enhanced neutralizing antibody titers when used as a priming dose prior to administration of a conditionally licensed adenovirus-vectored FMD vaccine. Further work is needed to develop these DNA plasmid-based constructs into standalone FMD vaccines in cattle.

## 1. Introduction

Foot-and-mouth disease (FMD) is a highly contagious vesicular disease affecting both domestic livestock, including swine and cattle, and wildlife. Foot-and-mouth disease virus (FMDV), the causative agent of FMD, is a member of the *Picornaviridae* family, and has an icosahedral capsid composed of four structural proteins, VP1, VP2, VP3, and VP4, derived from the processing of the FMDV P1 polypeptide by viral 3C protease [1,2,3]. FMDV capsid production in infected, [4], and transfected cells, [5,6,7,8], can result in crystalline arrays detectable by electron microscopy, and intact capsids are required to produce protective immunity against FMDV [9].

In endemic countries, FMD can be controlled through the vaccination of susceptible species. The most widely utilized vaccine is derived from a live virus that has been inactivated. Although effective, manufacturing requires the growth of large volumes of infectious virus requiring high-containment facilities for vaccine production and carries the risk of accidental escape. To avoid the need to culture live virus recombinant, FMD vaccines have been developed. Recombinant FMDV vaccine platforms use FMDV 3C protease to process the P1 polypeptide to produce virus like particles (VLPs) [8,10]. However, wild-type FMDV 3C protease activity in host cells also results in deleterious effects through degradation of multiple host proteins [11,12,13] which may limit the antigen yield and vaccine potency. One reported approach to limit deleterious effects is to reduce 3C concentrations in host cells through incorporating the HIV-1 ribosomal frameshift regulatory element [10]. Other approaches include mutating the 3C protease sequence to reduce off-target degradation of host proteins, such as L127P [5,7] and C142T [10]. However, reduced 3C activity is not essential to produce an effective vaccine as the replication-deficient human adenovirus serotype 5 (Ad5) FMDV vaccine uses an unmodified 3C protease [14,15,16].

The utility of nucleic acid-based vaccines as a versatile platform to express protective antigens has been proven with the success of mRNA-based vaccines against SARS-CoV-2. A DNA-based vaccine platform would offer flexibility in production with the potential advantages of greater stability and less stringent storage requirements, extended shelf-life, and prolonged duration of expression in vivo [17]. Previous studies of DNA vaccines against FMDV yielded mixed results, including one that protected cattle using two doses of DNA vaccine [18], but no treatment group in that study achieved 100% protection. More recent work using DNA plasmids expressing the P1 polypeptide and a mutant 3C protease found a significant increase in antigen yield [7], as well as the ability to manufacture FMDV VLPs for administration as a protective vaccine in both cattle and swine [8]. In this report, we evaluate both traditional DNA plasmid and DNA minicircle constructs, with and without mutant FMDV 3C sequences, for P1 processing and VLP formation in vitro, and as FMDV vaccine candidates after direct administration in cattle.

Minicircles are small plasmids lacking prokaryotic propagation elements [19], that are associated with negative effects on transfection efficiency and duration of transgene expression in mammalian cells [20,21,22,23,24,25,26]. Lab-based methods to design and produce minicircles include bacteriophage λ integrase [23], Cre recombinase [24,27], φC31 integrase [19,28,29], and ParA resolvase [30]. In this study, minicircles were produced using a commercial minicircle production system employing inducible φC31 integrase and Sce-I endonuclease expression [19,28].

All constructs in this study encoded the FMDV serotype O1 Manisa P1, but otherwise differed either in plasmid size or 3C protease yields. The pTarget O1P1-3C plasmid utilizes a wild-type 3C protease previously demonstrated to fully process the P1 polypeptide and produce VLPs in transfected cells [6]. The same O1P1-3C transgene construct was also used for the minicircle O1P1-3C construct.

Both the O1P1-HIV-3C^T^ minicircle and mpTarget O1P1-3C^LT^ plasmid constructs were designed to reduce negative effects on host cells using different methodologies. The O1P1-HIV-3C^T^ construct encodes an HIV-1 ribosomal frameshift with a C142T mutant 3C protease similar to constructs demonstrating successful production of FMDV VLPs in a baculovirus system [10]. The mpTarget O1P1-3C^LT^ plasmid encodes a 3C protease with two mutations, L127P and C142T, and is similar to plasmids used to produce a purified-VLP based vaccine candidate utilizing transiently transfected mammalian cells [7,8].

All constructs produced fully processed VP1-4 structural proteins in vitro, and crystalline arrays of VLPs were observed for constructs pTarget O1P1-3C, mpTarget O1P1-3C^LT^, and the O1P1-3C minicircle. None of the DNA vaccine constructs protected cattle from clinical FMD when used independently in a prime-boost regime. However, a priming dose of traditional plasmid pTarget O1P1-3C followed by the conditionally licensed Ad5 FMD vaccine resulted in enhanced FMDV neutralizing titers (VNT) compared to cattle receiving only the Ad5 FMD vaccine.

## 2. Materials and Methods

### 2.1. Construction of Plasmid Constructs

The plasmid constructs utilized in this report, pTarget and mpTarget, were constructed as previously described [7]. The pTarget and mpTarget plasmids differ in the presence of the Neomycin selection marker. In brief, P1-3C sequences were synthesized by Genscript and cloned into the pTarget (Promega, Madison, WI, USA) or mpTarget vectors utilizing restriction enzymes BamHI-HF^®^ and EcoRI-HF^®^ (New England Biolabs, Ipswich, MA, USA) as per the manufacturer’s instructions. Ligation was performed using T4 DNA Ligase (Roche, Indianapolis, IN, USA) and subsequently transformed into NEB^®^ 5-alpha competent *E. coli* (New England Biolabs) and plated on 50 µg/mL Carbomycin LB agar plates (Teknova, Hollister, CA, USA). Selected colonies were grown in imMedia™ Growth Medium with Carbomycin (Invitrogen, Waltham, MA, USA) overnight at 37 °C. Plasmids were isolated using QIAprep Spin Miniprep Kit (Qiagen, Redwood City, CA, USA) as per the manufacturer’s protocols. Mutation of the 3C sequence in the mpTarget O1P1-3C^LT^ plasmid was performed utilizing the GeneArt site-directed mutagenesis system (Invitrogen) with the previously reported primers [7].

### 2.2. Construction of Minicircle Plasmid Vectors

#### 2.2.1. Construction of pMC O1P1-HIV-3C^T^

Minicircle vector pMC.CMV-MCS-SV40polyA (System Biosciences, Palo Alto, CA, USA) and plasmid pUC57 O1P1-HIV-3C^T^, synthesized by Genscript and containing the O1 Manisa P1 sequence, were digested with BamHI-HF^®^ and EcoRI-HF^®^ (New England Biolabs) as per the manufacturer’s instructions. Ligation was performed using T4 DNA Ligase (Roche) and subsequently transformed into NEB^®^ 5-alpha competent *E. coli* (New England Biolabs) and plated on 50 µg/mL Kanamycin LB agar plates (Teknova). Selected colonies were grown in imMedia™ Growth Medium with Kanamycin (Invitrogen) overnight at 37 °C.

#### 2.2.2. Construction of pMC O1P1-3C

The pMC O1P1-3C plasmid was produced by digestion of the pMC O1P1-HIV-3C^T^ plasmid with NotI-HF^®^ and EcoRI-HF^®^ (New England Biolabs) according to the manufacturer’s instructions. The nucleotide sequence encoding the FMDV 3C was derived by PCR amplification of a template plasmid containing the coding region of FMDV Asia Lebanon 1989 strain (GenBank accession no: JF739177). Ligation was performed using T4 DNA Ligase (Roche) and subsequently transformed into NEB^®^ 5-alpha competent *E. coli* (New England Biolabs) and plated on 50 µg/mL Kanamycin LB agar plates (Teknova). Selected colonies were grown in imMedia™ Growth Medium with Kanamycin (Invitrogen) overnight at 37 °C.

#### 2.2.3. Construction of pMC SGLuc

Previously constructed pTarget super-luminescent Gaussia princeps luciferase (SGLuc) plasmid [6] was used as a template for the insertion of SGLuc into pMC.CMV-MCS-SV40polyA with BamHI-HF^®^ and EcoRI-HF^®^. Ligation was performed using T4 DNA Ligase (Roche), and subsequently transformed into NEB^®^ 5-alpha competent E. coli (New England Biolabs) and plated on 50 µg/mL Kanamycin LB agar plates (Teknova). Selected colonies were grown in imMedia™ Growth Medium with Kanamycin (Invitrogen) overnight at 37 °C.

### 2.3. Minicircle Production and Mammalian Cell Transfection

Minicircles were produced using the MC-Easy™ Minicircle DNA production kit (System Biosciences) following the manufacturer’s instructions. HEK293-T cells were transfected with 4 μg of plasmid vectors using Lipofectamine 2000™ (Life Technologies, Waltham, MA, USA) as per the manufacturer’s instructions and incubated for 24 h at 37 °C with 5% CO_2_ prior to cell harvest.

To monitor expression over time, porcine kidney IBRS2 cells were transfected, as described above, and incubated at 37 °C with 5% CO_2_ for up to 72 h post-transfection. To quantify gene expression every 24 h, the growth media (1× MEM, 10% fetal bovine serum, 5% 100× Antibiotic-Antimycotic) was removed completely; cells were rinsed with 1× dPBS and replenished with fresh growth media.

### 2.4. Evaluation of Transgene Expression, P1 Processing, and VLP Formation

Transfected HEK293-T cell lysates were harvested from 6-well plates (Corning, Glendale, AZ, USA) using 250 μL of M-PER™ Mammalian Protein Extraction Reagent (Invitrogen), loaded onto NuPAGE™ Novex™ 4–12% Bis-Tris protein gels (Invitrogen), and proteins transferred to nitrocellulose membranes using the iBlot^®^2 Dry Blotting System (Life Technologies). Recombinant protein expression was evaluated by western blot using detection monoclonal antibodies (mAb) F1412SA [31] for VP0 and VP2, and 12FE9.2.1 mAb for VP1 [32], as previously described [7]. Cytoplasmic VLP formation was evaluated by either transmission electron microscopy (TEM), as previously described [7], or by immune-EM utilizing gold labeled F1412SA antibody, as previously described [6].

For confirmation of VLP formation HEK293-T, cells were lysed using the cytosolic lysis buffer from the Qproteome Cell Compartment kit (Qiagen) with protease inhibitor added, as suggested by the lysis buffer manufacturer. The supernatant was removed and purified with a 10 mL 40 kDa MWCO Zeba Spin desalting column (ThermoFisher Scientific, Waltham, MA, USA), as suggested by the manufacturer, utilizing PBS as the exchange buffer. The resulting flow through was then applied to a 1,000,000 MWCO Vivaspin 20 centrifugal concentrator (Viva products, Littleton, MA, USA) and centrifuged at 2000× *g* for 5 min. The retained sample was used for immunizations or cesium chloride gradients.

#### 2.4.1. Cesium Chloride Gradient Analysis

Cesium Chloride gradient centrifugation was used for in-vitro analysis. Briefly extracted antigen was layered on top of 2 mL of 1.42 g/cm^3^ and 2 mL of 1.38 g/cm^3^ gradients prepared in TEN buffer (0.05 M Tris, 0.15 M NaCl, 0.001 M EDTA, pH 7.4). Gradients were centrifuged at 35,000 rpm for 18 h using an Optima L-80 XP ultracentrifuge (Beckman Coulter, Brea CA, USA). After centrifugation, visible bands were removed and dialyzed against PBS at 4 °C utilizing 10 K MWCO Slide-A-Lyzer Dialysis Cassettes (Thermo Fisher, Waltham, MA, USA).

#### 2.4.2. Immunogenicity in Guinea Pigs

Prior to utilization, the approval of animal use and study was obtained from the Plum Island Animal Disease Center Institutional Review Board and the Institutional Animal Care and Use Committee. Mixed-gender Hartley Guinea Pigs of less than 350 g in weight were obtained (Charles River Labs, Wilmington MA, USA) and allowed to acclimate for 7 days prior to any procedures being performed. Antigen was prepared by mixing 220 µg of total protein, as determined by Bradford assay, with Montanide ISA61VG (Seppic, Courbevoie, France) in a 1:1 ratio by volume. A final injection volume of 200 µL was used for each dose. Guinea Pigs were sedated for all procedures. Blood collection was performed prior to inoculation and every seven days after that till the end of the study at 28 days post-vaccination. All blood collections prior to 28 days post-vaccination utilized BD Microtainer Serum Separator Tubes (4MD Medical). At 28 days post-vaccination, Guinea Pigs were euthanized by cardiac puncture, and blood collected in serum separator tubes for future usage.

### 2.5. Cattle Vaccination and FMDV Challenge

Prior to conducting animal work, approval was obtained from the Plum Island Animal Disease Center Institutional Review Board and the Institutional Animal Care and Use Committee. Sixteen Holstein steers were administered one of five treatments. All DNA vaccines were mixed with TurboFect™ in vivo transfection reagent (Thermo Fisher), as per the manufacturer’s instructions, for primary inoculation prior to administration. Six cattle received human Adenovirus 5 vectored O1Manisa87.F (RGD).11D vaccine (Ad5O1M) [33] at a dosage 1 × 10^9^ plaque-forming units 14 days prior to challenge.

Blood samples were collected every seven days and evaluated for the presence of FMDV-neutralizing titers. Cattle were challenged intradermolingually with 10^4^ 50% bovine infectious doses (BID_50_) of FMDV O1 Manisa at 14 days post boost (dpb), and monitored for clinical FMD for 10 days, defined as the presence of vesicular lesions on one or multiple feet [33].

### 2.6. Testing Serum for Virus Neutralizing Antibody Titers

Neutralizing antibody titers against FMDV O1 Manisa was determined by virus neutralization test on BHK-21 cells following World Organization for Animal Health (OIE) protocols. Briefly, serum samples were heat-inactivated at 56 °C for 1 h and tested by incubation of 4-fold serial dilutions of serum with 100 TCID50 of FMDV for 1 h, followed by the addition of BHK-21 cells in 96-well plates and incubation at 37 °C and 5% CO_2_ for 72 h. Wells were examined under the microscope for cytopathic effect, and neutralization titers were expressed as the log_10_ of the reciprocal of the highest serum dilution resulting in 50% neutralization of the wells utilizing the Spearman-Karber method.

## 3. Results and Discussion

**Figure 1:**
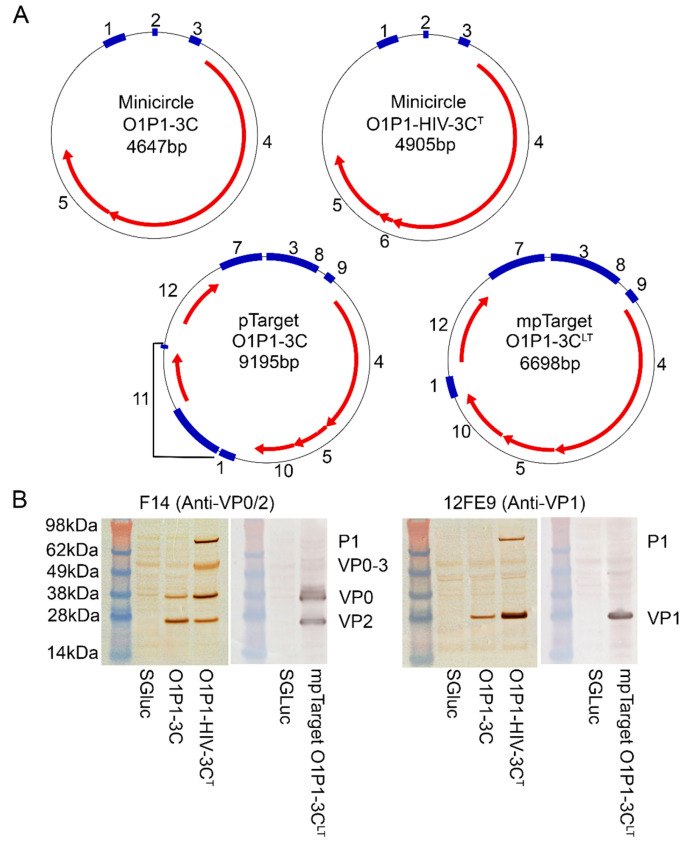
(**A**) All DNA vaccine constructs consisted of circular double-stranded DNA. Traditional plasmids, pTarget O1P1-3C and mpTarget O1P1-3C^LT^, are of larger size than minicircles O1P1-3C or O1P1-HIV-3C^T^. Minicircle and plasmid features; 1: SV40 polyA, 2: attP/attB, 3: CMV promoter, 4: O1P1, 5: 3C protease, 6: HIV frameshift, 7: ORI, 8: CMV enhancer, 9: Intron, 10: SGLuc biomarker, 11: Neomycin selection marker, and 12: Amp^R^. (**B**) Western blots of transfected cell lysates demonstrate fully processed VPs with O1P1-3C minicircles and mpTarget O1P1-3C^LT^ plasmid, whereas lysates of the O1P1-HIV-3C^T^ minicircle transfected cells demonstrate both fully processed VPs and partially processed intermediates.

Figure 1A shows the four DNA vaccine constructs including two minicircle-based constructs (O1P1-3C and O1P1-HIV-3C^T^) and two traditional plasmid-based constructs (pTarget O1P1-3C and mpTarget O1P1-3C^LT^). Minicircle constructs typically have fewer base pairs than plasmid constructs and lack prokaryotic propagation elements which can hinder transgene expression [17]. The difference in total base pairs between pTarget O1P1-3C and mpTarget O1P1-3C^LT^ is due to the lack of a Neomycin selection marker in mpTarget.

To confirm FMDV P1 expression and processing prior to vaccination, transfected HEK-293-T cell lysates were examined by western blot, Figure 1B. Lysates from O1P1-3C minicircle transfected cells showed 3C-dependent processing of P1 cleavage sites with VP0 staining less intense than the VP2 signal, suggesting a relatively higher amount of mature VP2 than unprocessed VP0 in the sample, Figure 1B. The strong VP2 signal indicates the assembly of intact empty capsid transfected cells, as VP2 is produced by autolytic cleavage of VP0 only after capsid assembly [34].

Cells transfected with O1P1-HIV-3C^T^ minicircles expressed higher levels of unprocessed P1 and partially processed VP0-VP3 intermediates, relative to both wild-type and 3C^LT^ levels, indicating that reduced 3C activity also reduced the FMDV P1 polypeptide processing efficiency in transfected cells. Cell lysates also had a higher VP0 signal relative to VP2, in contrast to O1P1-3C results (Figure 1B).

**Figure 2:**
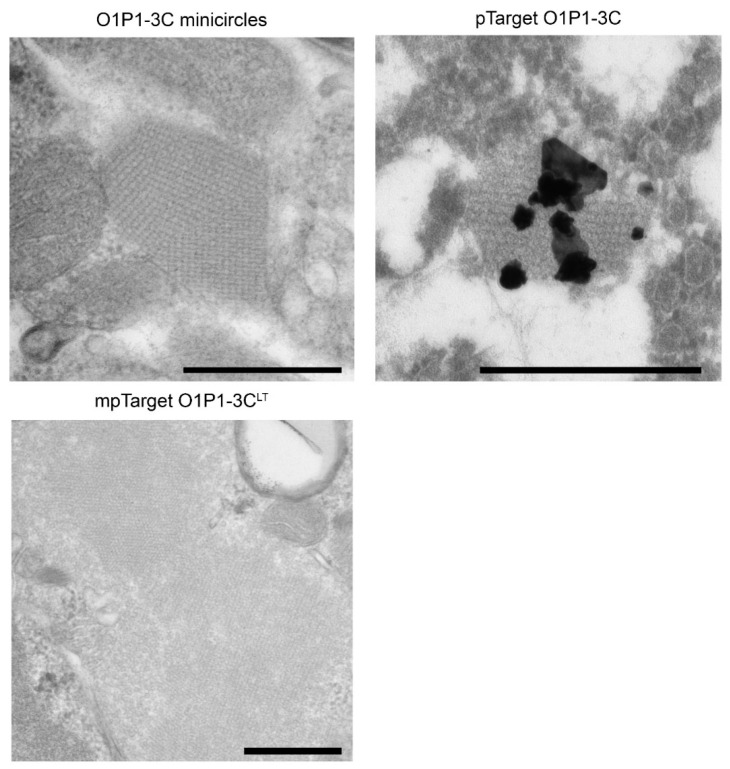
TEM of cells transfected with either O1P1-3C minicircles, pTarget O1P1-3C, or mpTarget O1P1-3C^LT^ plasmid, showing FMDV VLP crystalline arrays; the black bar represents 500 nm. Cells transfected with pTarget O1P1-3C were evaluated by immuno-EM utilizing gold labeled F1412SA antibody.

Traditional FMDV vaccines require the administration of intact capsids to ensure the presentation of structural viral epitopes to the immune system [35]. Our TEM analysis showed production of crystalline arrays of VLPs in HEK-293-T cells transfected with O1P1-3C minicircles, pTarget O1P1-3C plasmid, or mpTarget O1P1-3C^LT^ plasmid, Figure 2. The TEM results demonstrate minicircle technology as a viable DNA-vectored platform for FMDV VLP expression, and confirm previous findings that pTarget O1P1-3C plasmid expression produces VLP arrays [6]. Although intracellular VLPs were not seen with the O1P1-HIV-3C^T^ minicircles, additional purification and concentration of these samples may improve VLP detection as previously reported [10].

**Figure 3:**
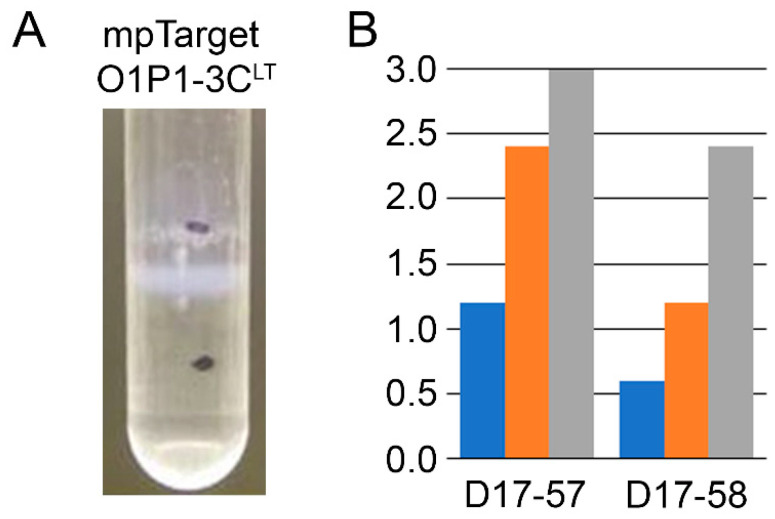
Antigen extracted from cells transfected with mpTarget O1P1-3C^LT^ plasmid was found to (**A**) sediment at the appropriate density using a cesium chloride density gradient and (**B**) produce VNTs in two guinea pigs, ear tags D17-57 and D17-58, inoculated in a non-challenge study at 7 (blue) and 14 (orange) days post-vaccination. VNTs were further enhanced at 28 days post-vaccination (gray) following a boost at day 21.

To further validate the presence of intact VLPs, mammalian cell cultures were transfected with the mpTarget O1P1-3C^LT^ plasmid and lysed for antigen extraction. The supernatant was evaluated by cesium chloride density gradient, to demonstrate proper sedimentation, and administration to guinea pigs in a non-challenge study. Extracted antigen was found to properly sediment, Figure 3A, and produce VNTs against O1 Manisa in guinea pigs, Figure 3B.

To evaluate the potential of these constructs as DNA vaccines, purified plasmids or minicircles were mixed with the in vivo transfection reagent TurboFect™ and administered to cattle in four treatment groups. A fifth treatment group received minicircles expressing SGLuc as a negative control. Serum FMDV neutralizing antibody titers were determined for all groups prior to the homologous challenge, Table 1. At the time of boost, 21 days post-vaccination (dpv), only three of the thirteen cattle administered DNA vaccine candidates showed FMDV serum VNTs above background levels; two cattle receiving O1P1-3C minicircles and one animal in the pTarget O1P1-3C plasmid.

Following a boost with either minicircle or plasmid, only one animal in the O1P1-3C minicircle group had increased VNTs post-boost, Table 1. Due to the low levels of VNTs observed at day 7 and 14 post-boost, two treatment groups were boosted with the Ad5 O1 Manisa vaccine (Ad5O1M), a vaccine with established efficacy in cattle, to evaluate if priming with a DNA vaccine enhanced neutralizing antibody response. All animals receiving the Ad5O1M boost demonstrated VNTs at 7 and 14 days post-boost (dpb). The geometric mean VNT titer of 1.6 log_10_ at 14 dpb in treatment group 3 was identical to previously reported results using the Ad5O1M vaccine alone under similar conditions [33].

Among all treatment regimes, cattle that received a pTarget O1P1-3C prime dose and an Ad5O1M vaccine boost dose had the highest geometric mean VNT titer (2.4 log_10_) at 7 dpb, suggesting that a DNA plasmid prime can enhance the host immune response following vaccination with Ad5O1M. Further exploration of combination DNA and Ad5 vaccines may provide enhancements to the Ad5 FMDV vaccine. After challenge, either 10 dpb for TG5, or 14 dpb for TG1-4, all cattle that received Ad5O1M (treatment groups 3 and 4) were protected from clinical FMD, whereas none of the cattle vaccinated with DNA plasmids alone were protected (treatment groups 1, 2, and 5) (Table 1).

**Figure 4:**
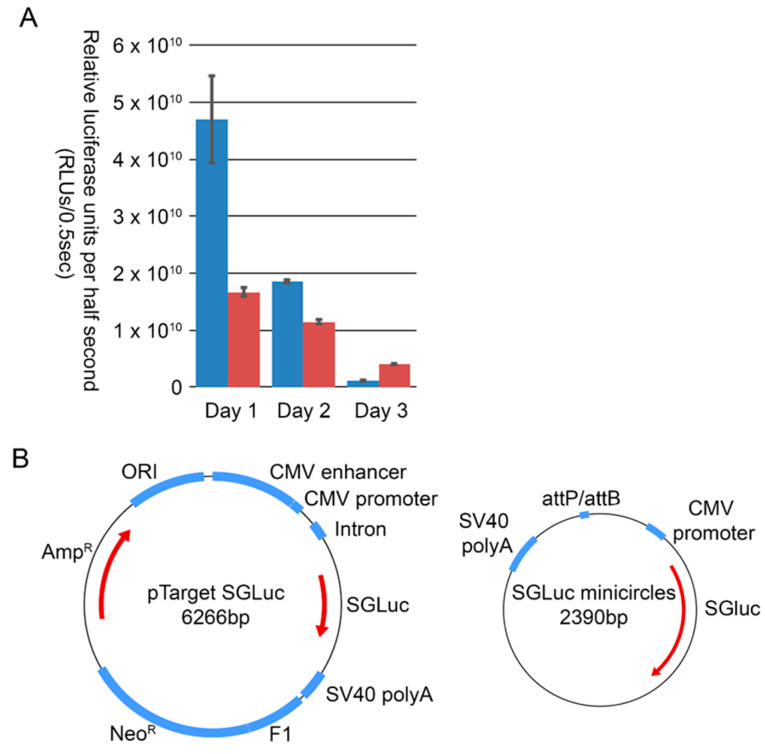
(**A**) Relative luciferase units per half second of IBRS2 cells transfected with either traditional plasmid, pTarget (blue), or minicircles (red), expressing the SGLuc reporter and monitored over three days of expression. (**B**) Minicircles are less than half the size of pTarget, they do not contain additional features to enhance transgene expression, such as chimeric intron and CMV enhancer sequences.

Despite the anticipated benefits to transgene expression from a minicircle backbone compared to other DNA vaccine platforms, we did not observe differences in vivo between the O1P1-3C minicircle, TG1, and conventional plasmid, TG4 and TG5. To evaluate if this observation was related to differences in transgene expression, minicircle and pTarget plasmids expressing only the SGLuc biomarker were tested in cell culture. Expression of pTarget SGLuc over the first 24 h was more than two-fold that of SGLuc minicircles in cell culture (Figure 4A). Expression from pTarget SGLuc continued to exceed that of SGLuc minicircles at 48 h post-transfection. Whereas minicircle transfected cell expression decreased at a lower rate over time, this benefit did not exceed the enhanced initial expression of the pTarget plasmid. One plausible explanation is associated with plasmid design. The minicircle plasmid contains only a CMV promoter whereas the pTarget plasmid contains a combination CMV promoter/enhancer sequence along with other plasmid features designed to enhance transgene expression (Figure 4B). This highlights the importance of plasmid design for transgene expression, and it is possible that alterations to components of DNA vaccine templates, such as the promoter-driving gene expression, may enhance the immunogenic response in cattle.

Minicircle O1P1-3C and plasmid pTarget O1P1-3C share similar transgene expression organization with the Ad5O1M vaccine despite divergent results in clinical protection. Although insufficient for protection against clinical disease, three DNA vaccine constructs elicited weak neutralizing antibody titers. The ability of mpTarget O1P1-3C^LT^ to produce extractable VLPs capable of eliciting neutralizing antibodies demonstrates that antigen produced from expression constructs retains the required epitopes for protection, Figure 3. VLPs extracted from transiently transfected cells using similar plasmids have been able to elicit protection from clinical disease following robust challenge in both swine and cattle [8]. This suggests that the antigen expressed from tested constructs can elicit a protective response under altered circumstances.

The lack of protection demonstrated herein is the likely resultant from either a failure to elicit sufficient expression, either through a lack of transfection efficacy or poor expression within transfected cells, a failure of antigen presentation following expression, such as through impaired VLP release, or a combination of factors. Although no treatment group receiving only DNA vaccines demonstrated protection it is possible that results may have differed if a less virulent strain, or less strenuous challenge methodology was applied.

## 4. Conclusions

An effective DNA plasmid-based vaccine platform for livestock would be a major technological advance, potentially enabling rapid and versatile vaccine optimization to combat specific FMDV outbreaks or epizootic strains. All of the DNA vaccine candidates in this study produced mature FMDV capsid proteins in cell culture but failed to elicit protection from clinical FMD in cattle under these study conditions. If extracted from transfected cell cultures, the VLPs are capable of eliciting neutralizing antibody titers. Technical hurdles must be overcome to develop the DNA vaccine constructs herein into an effective vaccine platform for FMD. Refinements such as improved plasmid backbones to enhance transgene expression, optimized dose and frequency, and more effective formulations and adjuvants have the potential to enhance the efficacy of these plasmid vectors as standalone DNA vaccines.

However, our results suggest that DNA vaccines used as a priming dose can enhance immune responses to subsequent Ad5-FMD vaccine administration. Production of plasmid-based DNA vectors is faster, easier, and cheaper than the production of Ad5-vectored vaccines, and using DNA plasmids in a prime-boost format may enhance response to Ad5-vectored vaccines through the broader or longer duration of immunity, or dose-sparing.

## 5. Patents

The work presented in this manuscript is contained within U.S. patents 9975926, 10513542, 10604548, 10858634, 10858933, and 10865389.

## Figures and Tables

**Table 1:** Average virus neutralization antibody titers against FMDV O1 Manisa in cattle.

TG	*n*	Prime	Dose	0 dpv	7 dpv	14 dpv	21 dpv	Boost	Dose	7 dpb	doc 14/10 dpb	Protected
1	3	MC O1P1-3C	0.5 mg	0.6	0.6	0.6	1.0	MC O1P1-3C	0.5 mg	0.8	0.8	0
2	2	MC O1P1-HIV-3C^T^	0.5 mg	0.6	0.6	0.6	0.6	None		0.6	0.6	0
3	3	MC SGLuc	0.5 mg	0.6	0.6	0.6	0.6	Ad5 O1 Manisa	1 × 10^9^ PFU	2.0	1.6	3
4	3	pTarget O1P1-3C	0.5 mg	0.6	0.6	0.6	0.8	Ad5 O1 Manisa	1 × 10^9^ PFU	2.4	2.1	3
5	5	mpTarget O1P1-3C^LT^	1.0 mg	0.6	0.7	0.7	0.6	mpTarget O1P1-3C^LT^	1.0 mg	0.6	0.6	0

Treatment group (TG); days post-vaccination (dpv); days post boost (dpb); day of challenge (doc). Assay has a 0.6 log_10_, limit of detection. TGs 1–4 were challenged 14 dpb whereas TG5 was challenged 10 dpb.

## Data Availability

The datasets used and/or analyzed during the current study are available from the corresponding author upon reasonable request.
